# Supplementary material for: Linear and phase controllable terahertz frequency conversion via ultrafast breaking the bond of a meta-molecule
Source: Nat Commun. 2024 Feb 6;15:1119. doi: 10.1038/s41467-024-45416-7 (PMC10847458; doi:10.1038/s41467-024-45416-7)
Supplement: Supplementary file 1 — Supplementary Information [file 41467_2024_45416_MOESM1_ESM.pdf]

Supplementary information

Linear and phase controllable terahertz frequency conversion via ultrafast breaking the bond of a meta-molecule

Siyu Duan[1,2], Xin Su[1,3], Hongsong Qiu[1], Yushun Jiang[1], Jingbo Wu[1,2*], Kebin Fan[1,2], Caihong Zhang[1,2], Xiaoqing Jia,[1,2] Guanghao Zhu,[1] Lin Kang,[1,2] Xinglong Wu[4,5], Huabing Wang[1,2], Keyu Xia[3,4,6*], Biaobing Jin[1,2*], Jian Chen[1,2], and Peiheng Wu[1,2]

[1]*Research Institute of Superconductor Electronics (RISE) & Key Laboratory of Optoelectronic Devices and Systems with Extreme Performances of MOE, School of Electronic Science and Engineering, Nanjing University, Nanjing 210023, China*

[2]*Purple Mountain Laboratories, Nanjing 211111, China*

[3]*College of Engineering and Applied Sciences, Nanjing University, Nanjing 210093, China*

[4]*National Laboratory of Solid State Microstructures, Nanjing University, Nanjing 210093, China*

[5] *School of Physics, Nanjing University, Nanjing 210023, China*

[6]*Shishan Laboratory, Suzhou Campus of Nanjing University, Suzhou 215000, China*

Correspondence should be addressed to Jingbo Wu (jbwu@nju.edu.cn), Keyu Xia (keyu.xia@nju.edu.cn), and Biaobing Jin (bbjin@nju.edu.cn)

This document contains supplementary information on "Linear and phase controllable terahertz frequency conversion via ultrafast breaking the bond of a meta-molecule." We provide details of the device fabrication, light path, and additional analysis results.

Pages S1-S22, 18 figures.

Supplementary Note 1. Device fabrication process

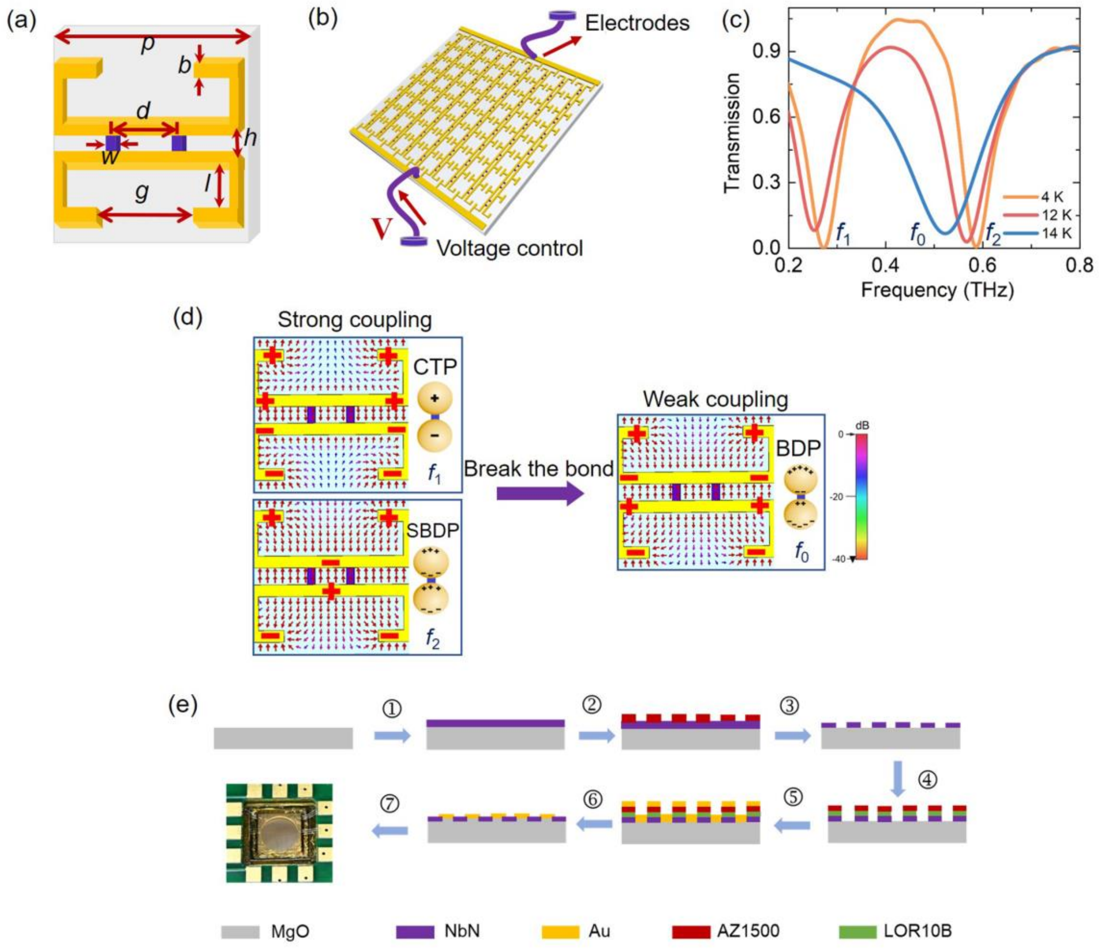

Fig. S1. Diagram and fabrication process of the hybrid metasurface. (a) Diagram illustrating the meta-molecule with the following geometrical parameters: w = 6 μm, b = 9 μm, h = 13 μm, l = 26 μm, d = 30 μm, g = 72 μm, and p = 120 μm. Adapted from supplementary Fig. S1a of supplementary Ref. 1. (b) Schematic diagram depicting the NbN-Au hybrid metasurface. (c) Simulated transmission spectra at different temperatures. (d) Simulated electric field distribution at the resonance frequencies of f_1, f_2, and f_0, corresponding to CTP, SBDP, and BDP mode, respectively. Adapted from supplementary Fig. S1d of supplementary Ref. 1. (e) Fabrication process flow of the NbN-Au hybrid metasurface.

We utilized electromagnetic simulation software to design and optimize the structure of the meta-molecule. The schematic of the NbN-Au hybrid metasurface is depicted in

Figs. S1a, b. The corresponding geometric parameters are as follows: w = 6 μm, b = 9 μm, h = 13 μm, l = 26 μm, d = 30 μm, g = 72 μm, and p = 120 μm. The polarization direction is perpendicular to the gap of the split ring resonator. The simulated transmission spectra and simulated electric field distribution at the resonance frequencies of f_1, f_2, and f_0 are presented in Fig. S1c, d. At 4 K, the NbN microbridges are in the superconducting state, and a strong conductive coupling exists between the upper and lower meta-atoms[1]. The transmission spectrum exhibits two resonant valleys corresponding to the charge transfer plasmonic (CTP) mode and the screened bonding dimer plasmonic (SBDP) mode in the plasmonic dimer[2-4].

When the temperature reaches 14 K, the NbN microbridge becomes lossy, suppressing the conductive coupling. In this case, there is only one resonance valley in the transmission spectra, corresponding to the bonding dimer plasmonic (BDP) mode[1-4]. The spectral responses in the superconducting and normal states are distinct, indicating the switching of the resonant modes. The simulated transmission spectra agree well with the calculated spectra (Fig. 1c). Furthermore, the kinetic inductance of the NbN microbridges increases with temperature, resulting in a tuning behavior observed in the transmission spectra[5].

The fabrication process of the device is outlined as follows:

(1) Depositing NbN film onto the MgO substrate.

(2) Spin-coating photoresist and UV photolithography.

(3) Reactive ion etching (RIE) to pattern the NbN film.

(4) Spin-coating photoresist and UV photolithography.

(5) RF sputtering of Au film followed by a lift-off process.

(6) Assembling the device on the printed circuit board and wire bonding.

Supplementary Note 2. THz Pump- THz probe spectroscopy system

The optical path diagram of a cryogenic THz pump-probe THz spectroscopy system is presented in Fig. S2. The femtosecond laser pulse has a repetition frequency of 1 kHz, an output power of 6 W, and a central wavelength of 800 nm. A 1-mm-thick ZnTe crystal

is employed to generate the THz probe pulses. The intense THz pump pulse is generated in a $LiNbO_3$ crystal using tilted-pulse-front optical rectification[6]. Another ZnTe crystal is utilized to detect the temporal waveform of the transmitted probe pulse based on free-space electro-optical sampling. The field strength of the THz pump pulse is adjusted using a wire grid polarizer (WG_1). WG_2 reflects THz probe pulses and transmitted pump pulses, while WG_3 is positioned behind the sample to block the THz pump pulse. The chopper is placed in the path of the laser beam to generate THz input signals. The chopper modulates the input signal with a modulation frequency of 370 Hz, while the THz pump wave is unmodulated. In the signal acquisition process, we utilized a lock-in amplifier to extract the modulated signal, ensuring that the inference from the THz pump is excluded from the measurements.

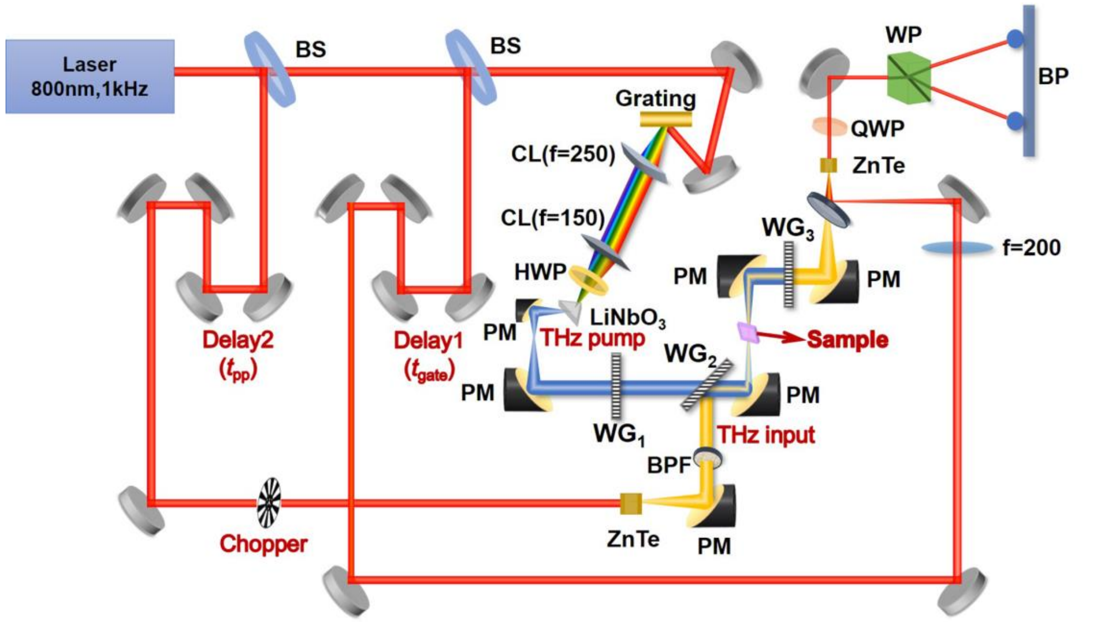

Fig. S2 Diagram of the THz pump-THz probe spectroscopy system: BS: Beam splitter, CL: Cylindrical lens, HWP: Half-wave plate, PM: Parabolic mirror, WG: Wire grid polarizer, QWP: Quarter-wave plate, WP: Wollaston prism, BP: Balanced photodiode, BPF: Metallic mesh bandpass filter. Adapted from supplementary Fig. S2 of supplementary Ref. 1.

Supplementary Note 3. Time-domain and frequency spectra of the THz pump pulse

For the THz pump-THz probe spectroscopy experiment, the time-domain profile and frequency spectrum of the THz pump pulses are plotted in Fig. S3a,c. The duration of the THz pump pulse is approximately 3 ps, and it has a bandwidth ranging from 0.2 to 1.2 THz. The maximum electric field strength of the THz pulse (E_0) reaches 25 kV/cm.

When only the pump pulse with the electric field direction perpendicular to the gap passes through the sample, the waveform of the transmitted pulse is plotted in Fig. S3b. The corresponding power spectrum is shown in Fig. S3c. As shown in Fig. S3d, the THz transmission spectrum is consistent with the calculated spectrum in the weak-coupling regime of Fig. 1c. It means the pump pulse can trigger the phase transition of superconducting microbridge from the superconducting state to the normal state, *i.e.*, "break the bond."

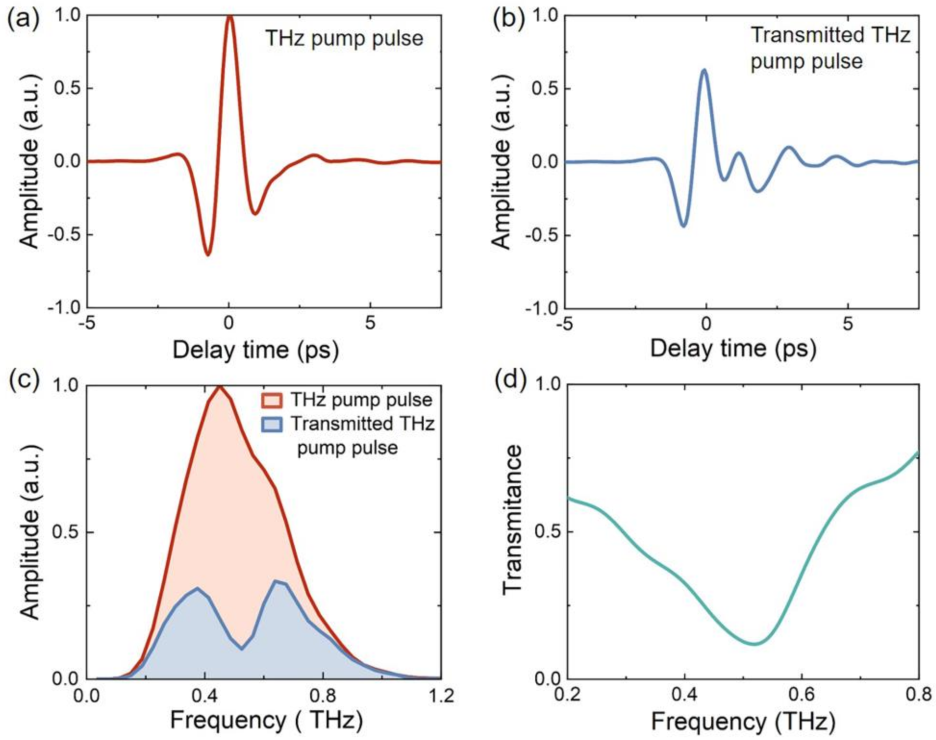

Fig. S3. Measured time-domain waveform of the pump pulse (a) and the transmitted pump pulse (b). (c) Corresponding power spectra of the input and transmitted pump pulses. (d) Transmission spectrum which is the ratio of the power spectrum of the transmitted pump pulse to the input pump pulse.

Supplementary Note 4. Calculated and measured THz probe pulse

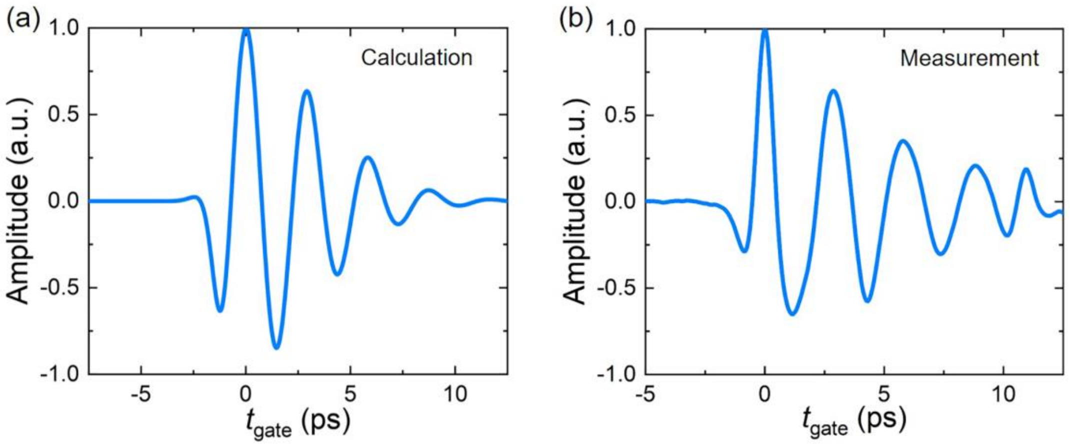

Fig. S4. Time-domain profile of the input narrowband THz pulse. (a) Calculated and (b) measured THz probe pulse.

The time-domain profiles of the calculated and measured narrowband input THz pulse are plotted in Fig. S4a, b. The width of the measured narrowband pulse is approximately 13 ps.

Supplementary Note 5. Transmitted THz pulse waveforms with 0.34 THz as the center frequency as a function of t_{gate}

The mapping of the electric field of the output pulse (E_{probe}) as a function of pump-probe delay (t_{pp}) is illustrated in Fig. S5a for an incident signal centered at 0.34 THz. Within the time range of -11.3 ps < t_{pp} < 0 ps, the tail oscillations of E_{probe} gradually occur earlier, as indicated by t_{gate}. The oblique blue line represents the trace where the pump and input pulse overlap[7]. In the range of -8.0 ps ≤ t_{pp} < -1.5 ps, new frequency components emerge outside the input spectrum. Notably, when t_{pp} = -3.7 ps, both the time and frequency domain spectra undergo significant changes. The time-domain waveforms of the output pulses at t_{pp} = -11.3, -8.0, -3.7, -1.5, and 0 ps are shown in Fig. S5b, corresponding to the black dashed lines marked in Fig. S5a.

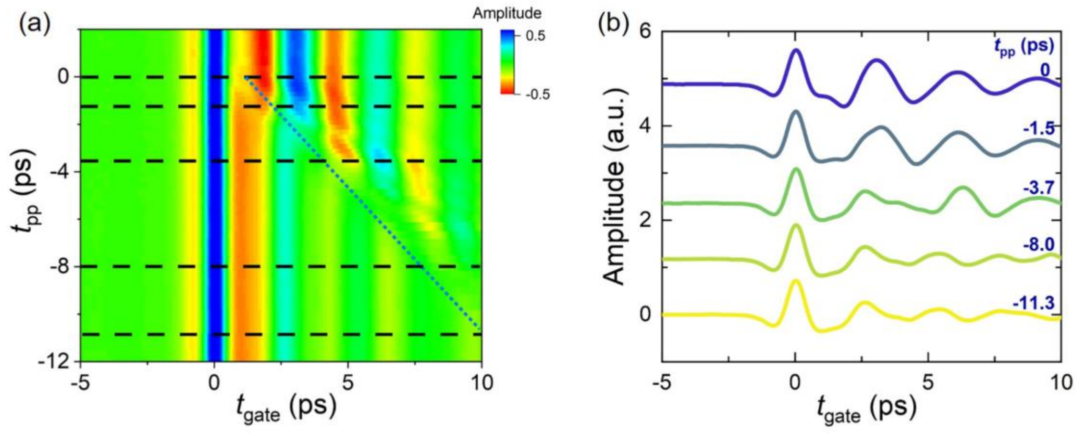

Fig. S5. Measured waveform of the output pulse with 0.34 THz as the center frequency. (a) Mapping of the electric field of the output pulse (E_{probe}) as a function of pump-probe delay (t_{pp}). The oblique blue line guides the eye to indicate the trace of the pump pulse. (b) Output pulse waveform at t_{pp} -11.3, -8.0, -3.7, -1.5, and 0 ps, corresponding to the black dashed lines marked in (a), respectively.

Supplementary Note 6. Frequency conversion at different pump-probe delays

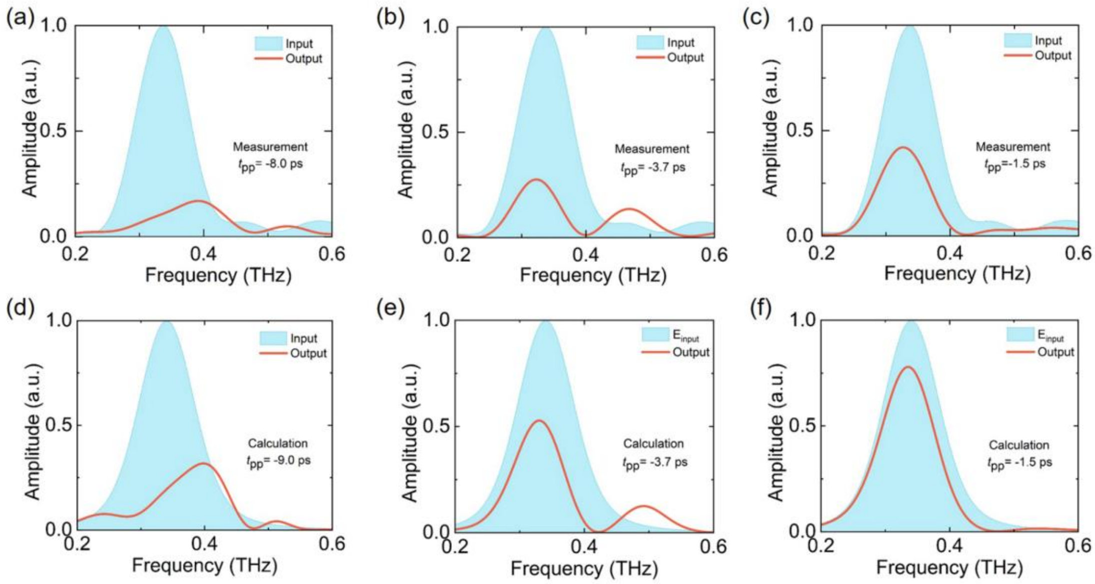

Fig. S6. Measured output and input power spectra (cyan shaded area) when t_{pp} = -8.0 ps (a), -3.7 ps (b), and -1.5 ps (c), and corresponding calculation results based on the coupled-mode model when t_{pp} = -9.0 ps (d), -3.7 ps(e), and -1.5 ps (f).

We utilized the transmission spectrum through the blank MgO substrate as a reference. The external frequency components outside the output power spectra first emerged during the time-varying process at t_{pp} = -8.0 ps. When the output power spectra were fully encompassed within the reference region, t_{pp} = -1.5 ps. Figs. S6a-c show the measured output power spectra at t_{pp} = -8.0, -3.7, and -1.5 ps. Based on the coupled-mode model, we calculated the output power spectra for t_{pp} = -9.0, -3.7, and -1.5 ps. In the model, the appearance of external frequency components beyond the output power spectra commenced at t_{pp} = -9.0 ps. As shown in Figs. S6d-f, the measured output power spectra exhibit good agreement with the calculated results.

Supplementary Note 7. Conversion efficiency as a function of t_{pp}.

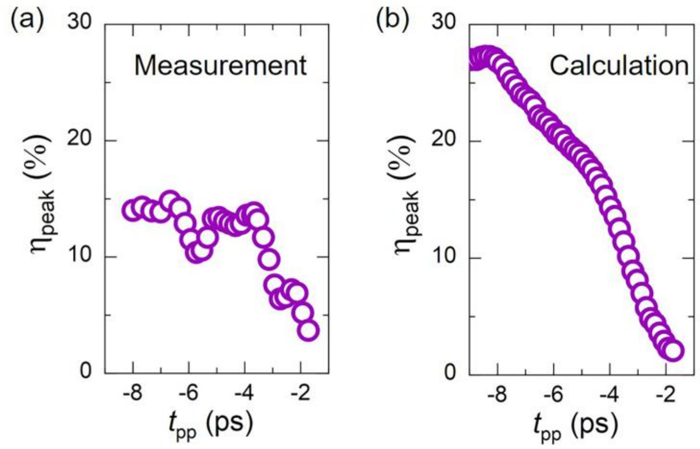

Fig. S7. (a) Measured and (b) calculated the efficiency of the conversion peak (η_{peak}) as a function of t_{pp}.

We calculated the efficiency of the conversion peak (η_{peak}) as the ratio of the peak power of the converted wave to the peak power of input wave[8]. Figures S7a,b show the measured and calculated efficiency of the conversion peak as a function of t_{pp}. The maximum efficiency of the conversion peak from measured and calculated spectra is 15% and 27%, respectively. During the time-varying process, the efficiency of the conversion peak gradually decreases as t_{pp} increases. The calculated and experimental results exhibit good agreement.

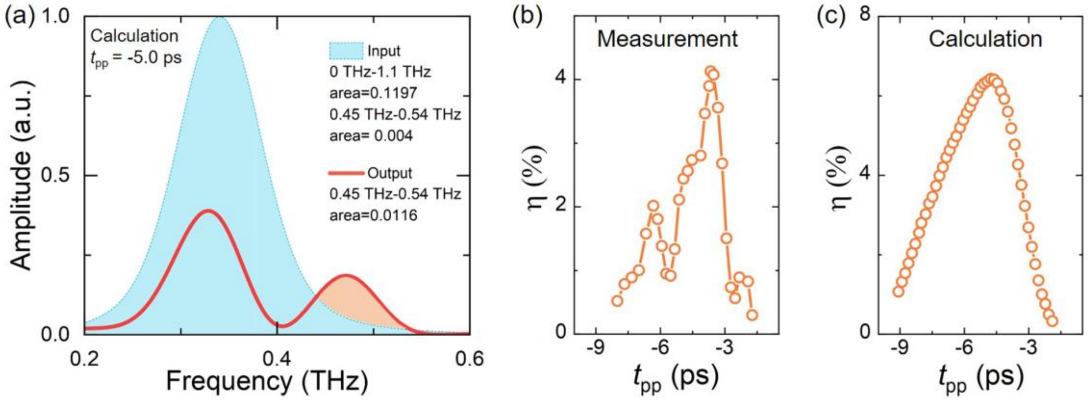

Fig. S8. (a) Calculated input and output power spectra when t_{pp} = -5.0 ps. The purple region indicates the output power spectrum beyond the input power spectrum. Conversion efficiency (η) as a function of t_{pp} calculated from the experimentally measured (b) and calculated (c) power spectra.

We also calculated the conversion efficiency (η) by integrating the frequency conversion components of the output power spectrum and the total input power. The purple region in Fig. S8a is the frequency conversion region minus the area within the corresponding frequency range of the input power spectrum. In this case, conversion efficiency is defined as the ratio of output power beyond the input power spectrum to the total input power. The calculated conversion efficiency is 6.37% when t_{pp} = -5 ps. Figures S8b,c show the conversion efficiencies obtained from the calculated and measured transmission spectra. They exhibit a similar trend as the t_{pp} increases. The maximum conversion efficiencies obtained from the calculated and measured transmission spectra are 6.4% and 4.1%, respectively.

Supplementary Note 8. Frequency conversion when the center frequency of the input signal is 0.6 THz.

We investigated the power spectra and conversion efficiency during the time-varying process by varying the input pulse frequency. In **Fig. 1c**, the transmission spectra exhibit two resonance valleys (f_1 and f_2) when the coupling interaction is strong. To experimentally and comprehensively verify the frequency conversion effect, we

utilized a bandpass filter with a center frequency of around 0.6 THz (approximately f_2).

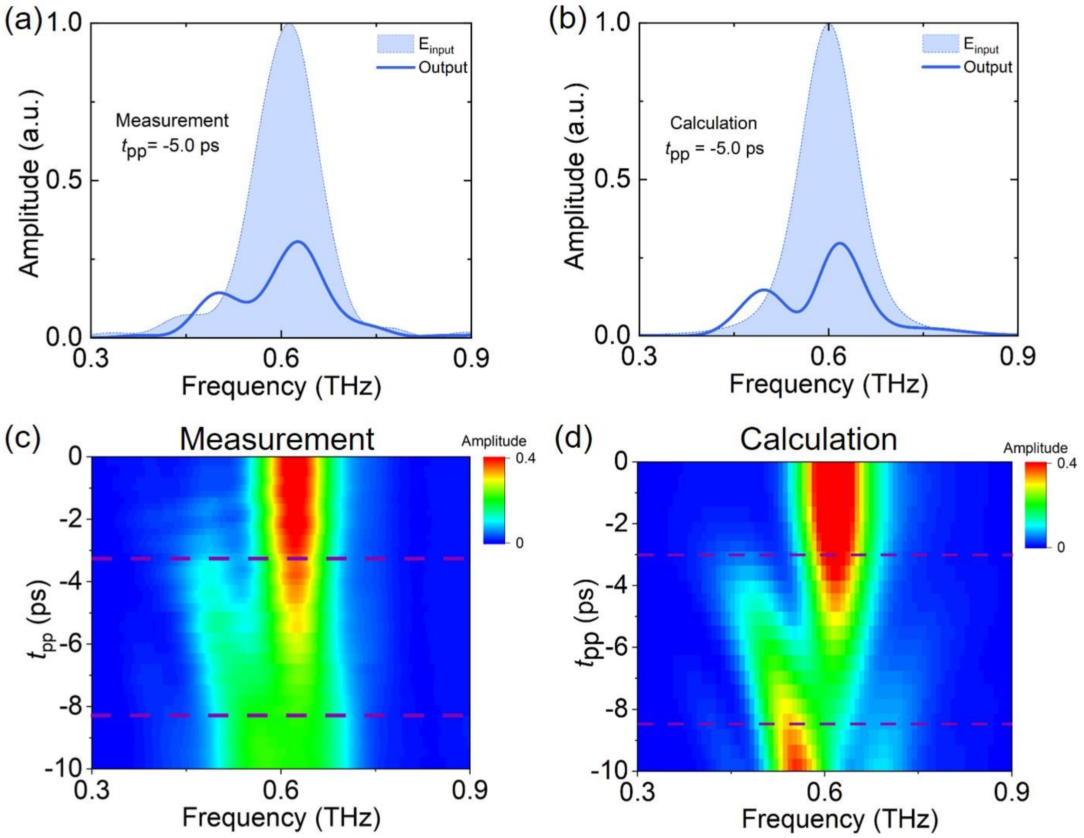

Fig. S8. (a) Measured and (b) calculated power spectra of the output signal at t_{pp} = -5.0 ps (blue line) when the center frequency of the input signal is 0.6 THz. The blue-shaded area represents the power spectrum of the input signal. Mapping of (c) measured and (d) calculated power spectra as a function of t_{pp}.

The output power spectrum through a blank MgO substrate was employed as the reference. The blue-shaded region in Fig. S8a indicates the bandwidth is approximately 60 GHz. The power spectrum for t_{pp} = -5.0 ps is depicted by the blue line in Fig. S8a, revealing a peak beyond the reference region on the left side of the spectrum. In Fig. S8b, the purple shaded area represents the input power spectrum obtained by setting f_{in} = 0.6 THz in the calculation model, while the blue line corresponds to the calculated output power spectrum at t_{pp} = -5.0 ps. Hence, frequency conversion is achieved.

The two-dimensional plot of the measured transmitted power spectra as a function of t_{pp} is presented in Fig. S8c. Within the range of $-8.2 \leq t_{pp} < -3.2$ ps, new frequency

components emerge beyond the input spectrum. The calculated power spectra with a time-varying response range are illustrated in Fig. S8d. According to the model, the additional frequency components outside the input power spectra appear at $-8.4 \leq t_{pp} < -3.0$ ps. The theoretical results agree well with the experimental findings.

Supplementary Note 9. Linear and tunable frequency conversion when the center frequency of the input signal is 0.6 THz

The power spectra were obtained under different input powers when $t_{pp} = -3.8$ ps, as shown in Fig. S9a. The conversion efficiency of the converted wave is determined at the peak (marked as A-D). Figure S9b shows the measurement data (circles), with a dashed line indicating the corresponding linear fit. The relationship between converted and input power follows a linear relationship[9].

The frequency and efficiency of the conversion peak at different t_{pp} in Figs. S8c, d are extracted and presented in Figs. S9c, d. The measured peak frequency of the conversion waves ranges from 0.51 THz to 0.48 THz, offering a frequency tuning range of approximately 30 GHz. The efficiency of the conversion peak reaches up to 15%. Additionally, the calculated peak frequency of the conversion waves gradually red-shifts from 0.52 THz to 0.46 THz, exhibiting a tuning range of approximately 60 GHz. The efficiency of the conversion peak shows a gradually decreasing trend with increasing t_{pp}. The maximum conversion efficiencies obtained from experiment and calculation are 1.3% and 2.6%, respectively. In contrast, the frequency tuning range and conversion efficiency of the calculated spectra are higher than the measured values. One possible reason for this discrepancy is the dispersive characteristic of the NbN film conductivity. As the frequency increases, the real part of the conductivity gradually decreases[10, 11]. In other words, the Ohmic loss of the superconducting microbridge at 0.6 THz is higher than that at 0.34 THz. Based on this analysis, the higher loss may lead to a reduction in the conversion efficiency.

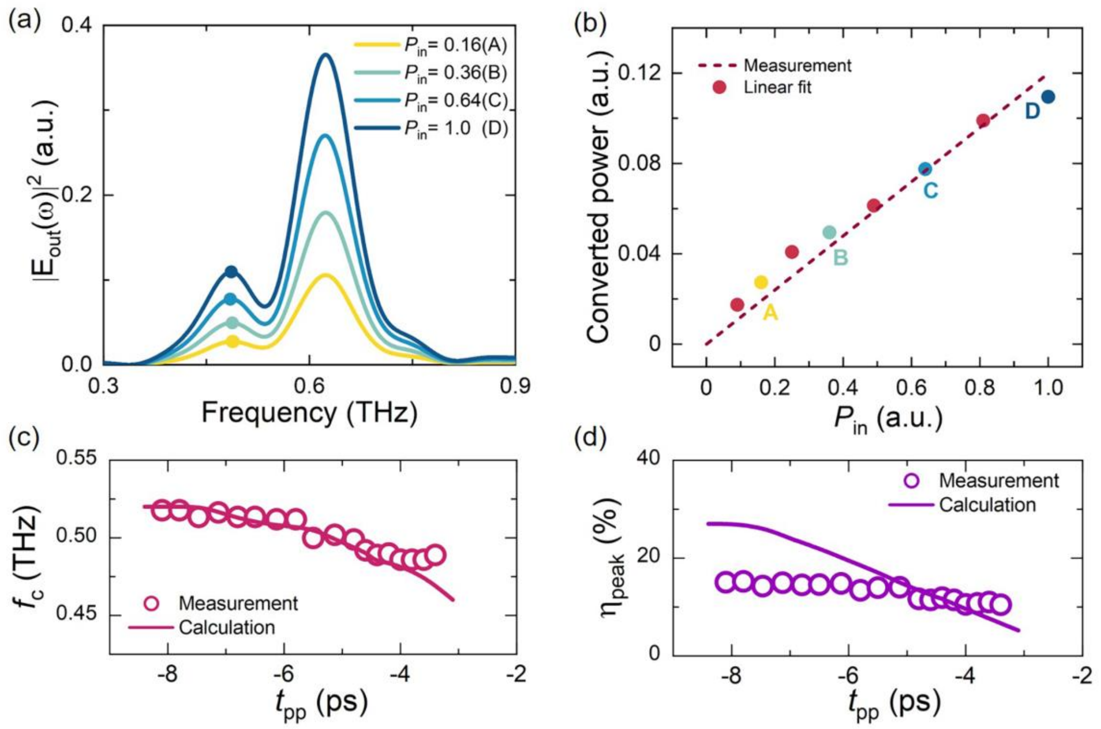

Fig. S9. Linear and tunable frequency conversion when the center frequency of input signal is 0.6 THz. **a**. Power spectra under different input power when t_{pp} = -3.8 ps. **b** Linear relationship between the input power and converted power at 0.48 THz. The linear fit is represented by a dashed line, while the measurement data are plotted with circles. **c** Frequency (f_c) and **d** efficiency of the conversion peak (η_{peak}) as a function of t_{pp}.

Supplementary Note 10. Electrically tuning of frequency conversion

The diagram of the hybrid meta-molecules with the electrical bias apparatus is illustrated in **Fig. S10a**. Each row of unit cells is connected in parallel, and the electrodes are linked to the electric source. When an electric bias is applied, current flows from one meta-atom, passes through the superconducting microbridges, and reaches the other meta-atom. Due to the thermal effect induced by the electric current, the superconducting microbridges become heated, and the coupling strength between two meta-atoms is changed, resulting in significant changes in the output spectra. Hence, the electric bias enables the alteration of the time boundary and the control over the time-varying response of the hybrid meta-molecules. The output power spectra under

various electrical powers (P_e) when t_{pp} = -3.7 ps were obtained and plotted in **Fig. S10b**. The electrical bias is observed to modulate the amplitude of the converted wave. In **Fig. S10c**, it is evident that the efficiency of the conversion peak at 0.47 THz gradually decreases with increasing P_e.

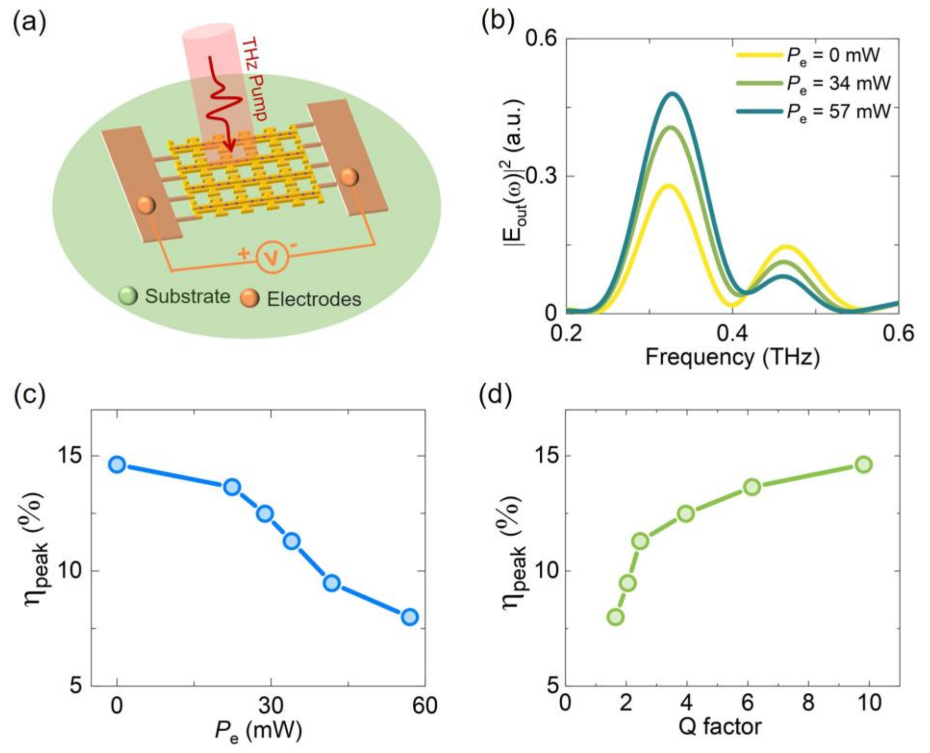

Fig. S10. Electrical tuning of frequency conversion. a) Schematic diagram illustrating electrically tunable THz frequency conversion. b) Measured output power spectra under different electric powers when t_{pp} = -3.7 ps. **c**) Efficiency of the conversion peak at 0.47 THz as a function of electric power. **d**) Efficiency of the conversion peak as a function of Q factors of the resonance mode at f_1.

From the THz transmission spectra obtained without the THz pump, we performed calculations to determine the quality factor (Q) of the resonant mode, which corresponds to f_1 in **Fig. 1c**, under various electric powers. The efficiency of the conversion peak as a function of the Q factors is shown in **Fig. S10d.** It is observed that higher Q factors correspond to higher amplitude of conversion peak. Additionally, we conducted temperature-dependent measurements of the conversion efficiency. Both the Q factor and the efficiency of the conversion peak decrease with the increasing

temperature. These results indicate that the loss of the metasurface is crucial in affecting conversion efficiency.

Supplementary Note 11. Measured transmission spectra without THz pump

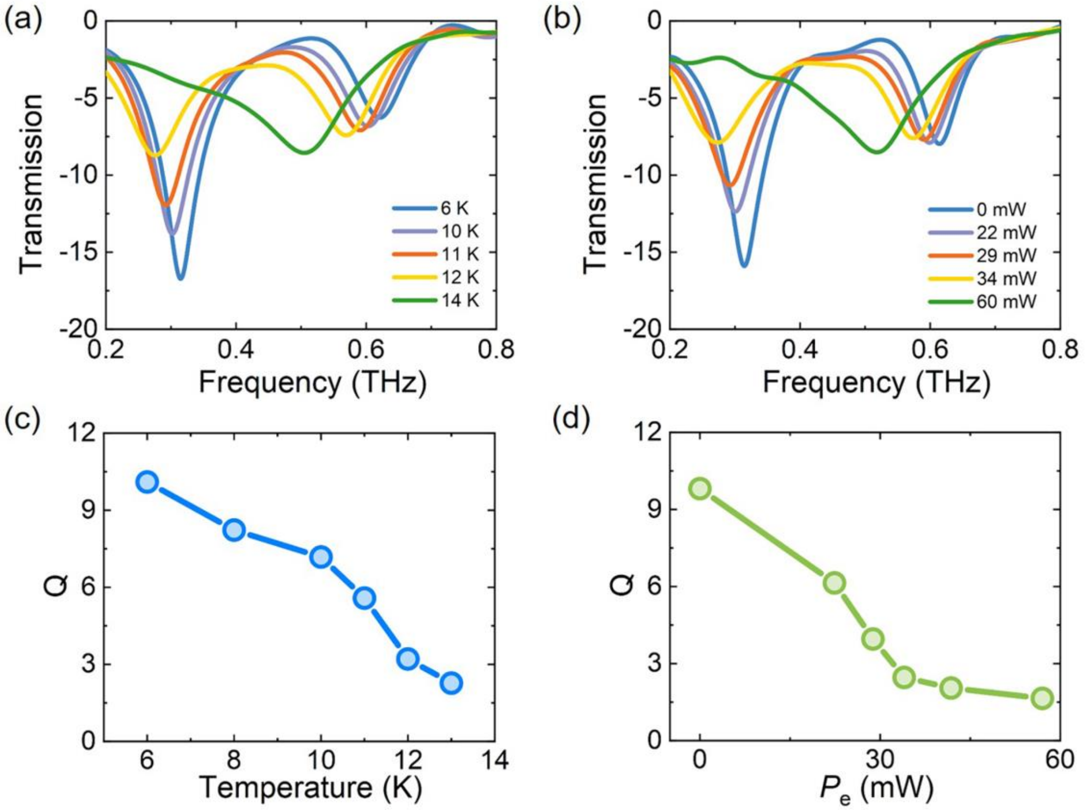

Fig. S11. Measured THz transmission spectra without THz pump. Measured THz transmission spectra under different temperatures (a) and electrical power (b). Q factors of the resonance mode around f_1 at various temperatures (c) and electrical powers (d).

The steady-state spectral response was measured with the incidence of a broadband THz probe pulse. At 4 K, we measured transmission spectra under different temperatures and electrical power, as shown in Figs. S11a, b. As the temperature and electrical power increase, the transmission spectra exhibit similar spectral response and tuning behaviors, agreeing well with the simulation spectra. The Q factor of the resonance mode was calculated as [12] $Q = f_0 / \Delta f$, and the extracted Q factors as a function of temperature and electrical power are presented in Figs. S11c, d. The Q factor

decreases as the temperature and electrical power increase.

Supplementary Note 12. Temperature tuning of THz frequency conversion

The output power spectra were obtained under different temperatures when t_{pp} = -3.7 ps, as shown in Fig. S12a. The conversion peak at 0.47 THz exhibits a decrease with increasing temperature, indicating temperature tuning of the converted wave. The efficiency of the conversion peak as a function of temperature is shown in Fig. S12b, illustrating a reduction in the efficiency of the conversion peak with increasing temperature. It can be attributed to the gradual increase of Ohmic loss caused by the suppression of superconductivity in the NbN microbridge as the temperature rises. These results highlight the impact of loss on the efficiency of frequency conversion.

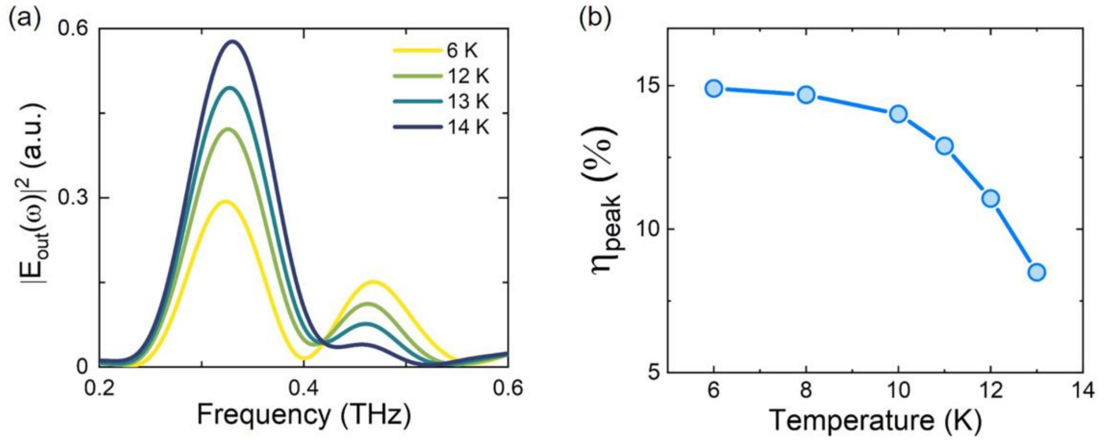

Fig. S12. Temperature tuning of THz frequency conversion**.** (a) Measured transmission power spectra under different temperatures when t_{pp} = -3.7 ps. (b) Efficiency of the conversion peak at 0.47 THz under different temperatures.

Supplementary Note 13. Extended research in topological dynamics and Floquet physics based on the proposed design

In our work, two meta-atoms are coupled by superconducting microbridges. The coupling strength (*J*) can be temporally tuned by an intense THz pulse at the picosecond level. By extending the design of two coupling meta-atoms to a 1D topological chain shown in Fig. S13 or a 2D topological metasurface shown in Fig. S14, and fast tuning

the coupling strength, we have the capability of studying the temporal dynamics using the topological metasurface.

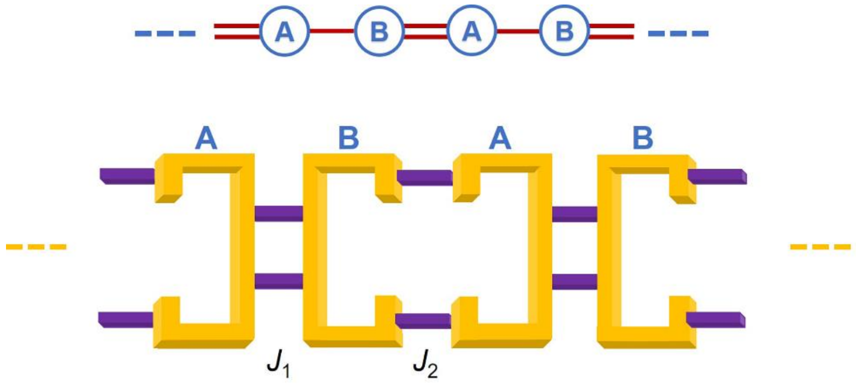

Fig. S13. A 1D chain of coupled meta-atoms for studying the topological dynamics of the SSH model.

The configuration shown in Fig. S13 creates a 1D chain of meta-atoms. We name the two coupled meta-atoms as A and B, respectively. In each unit cell, the meta-atoms of A and B are coupled with a rate of J_1. The strength of the A-B coupling between the unit cells is J_2. Then, the meta-atoms form a standard Su-Schrieffer-Heeger (SSH) model. Although the topology of the SSH model has been investigated in the metasurface platforms, the topology-related temporal dynamics in the THz regime are rarely reported.

The configuration in Fig. S14 provides a possible design for 2D topological metasurface[13]. The topology feature is crucially dependent on the three coupling rates: J_1, J_2, and J_3, where J_3 denotes the coupling between the upper and lower meta-atoms. We can change the coupling ratios in time by fast-tuning the coupling rates. Then, the temporal transition between topological phases with different Chern numbers and the dynamic emergence of the edge and corner states can be investigated in the two configurations.

In this work, the two meta-atoms are coupled via superconducting microbridges. This coupling can be switched off within a few picoseconds with a THz pulse and restored on a nanosecond scale. Thus, by applying a series of THz pulses with a temporal period (T), we can periodically modulate meta-atom couplings at a specific

frequency (1/*T*). It means that we create a periodic Hamiltonian to describe the system. By doing so, we create Floquet metamaterials and introduce a "synthetic" dimension to the system. Hence, we can study Floquet physics, such as forming Wannier-Stark ladders[14]. If we periodically modulate the coupling in the topological metamaterials, as shown in Figs. S13, S14, we can reveal topological Floquet physics.

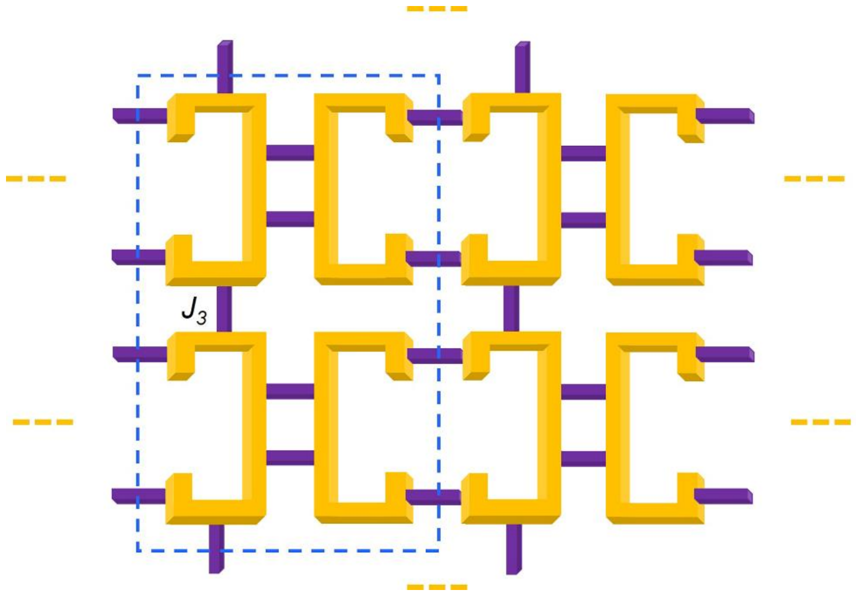

Fig. S14. A 2D metasurface consisting of coupled meta-atoms for studying the topological dynamics of 2D topological THz photonics.

Supplementary Note 14. Extended research in on-chip THz linear frequency converter based on the proposed design

One envisioned application for designing time-dependent metasurfaces is to achieve on-chip frequency conversion. The schematic of on-chip frequency conversion based on a single time-varying meta-molecule is shown in Fig. S15. For guiding the electromagnetic waves, we chose a single planar wire transmission line that lies on top of a supporting dielectric substrate, known as a planar Goubau line (PGL)[15]. The coplanar waveguide (CPW) couples electromagnetic energy into and out of the PGL. The designed meta-molecule interacts with the electric field of the PGL. Compared to the meta-molecular arrays, this approach significantly reduces the energy required to drive the phase transition by three orders. It can simplify the measurement setup and

make it more accessible. By controlling the phase transition of the single meta-molecule, the frequency conversion can be achieved.

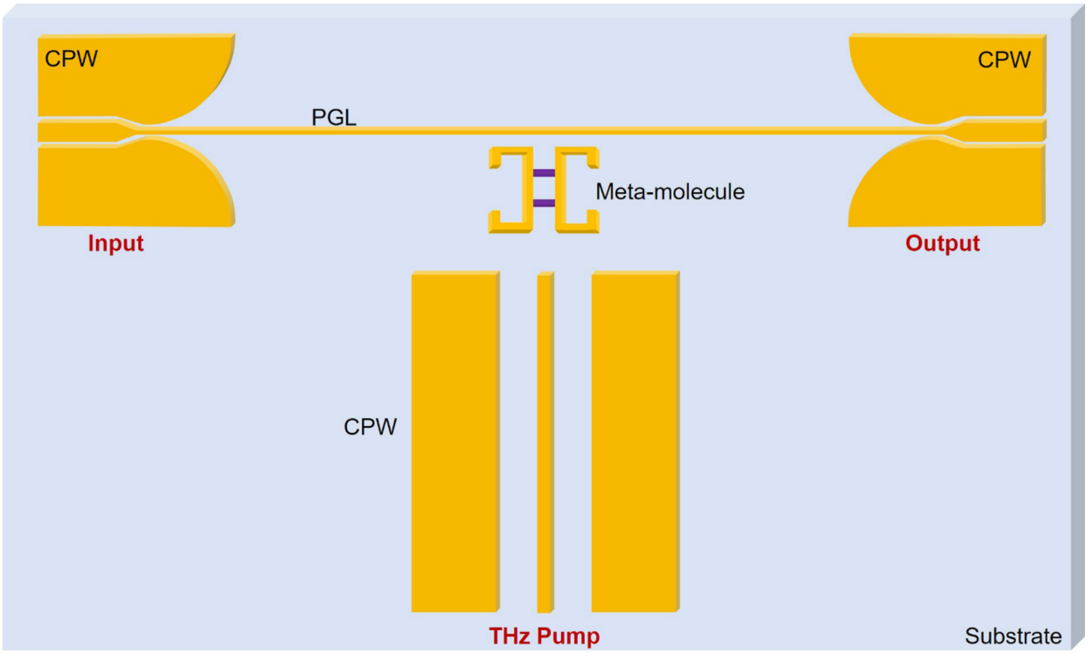

Fig. S15. On-chip frequency conversion based on single time-varying meta-molecule.

Supplementary Note 15. The relationship between the conversion efficiency and the frequency shift of converted wave

The relationship between the conversion efficiency and the relative frequency shift is a valuable topic. Since the resonance frequency of the two identical meta-atoms is as high as 0.48 THz, the frequency conversion at the frequency of high-order modes was not observed. Because the resonant frequency of meta-atoms without coupling is not tunable, it is difficult to study the relationship between conversion efficiency and frequency shift for different-order modes in experiments as in the previous work[16].

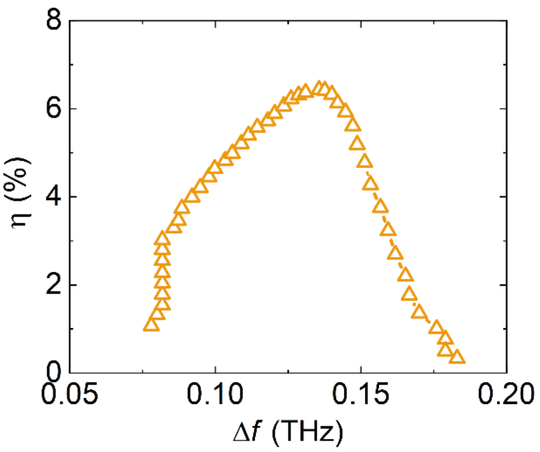

Fig. S16 Calculated conversion efficiency (η) versus frequency shift (Δf) under different t_{pp} when $|J|_{max}$ = 0.167 THz.

In our work, the frequency shift of the converted wave (Δf) and the conversion efficiency (η) can be adjusted by changing the time delay (t_{pp}). We first analyzed the relationship between Δf and η under different t_{pp} when $|J|_{max}$ = 0.167 THz. The relationship of calculated η versus Δf is shown in Fig. S16. Here, $\Delta f = f_c - f_{in}$, where f_c is the peak frequency of the converted wave, and f_{in} is the center frequency of the input wave. The value of η first increases with the increasing Δf, and reaches a maximum of 6.4% at a Δf of 0.135 THz. Then, η gradually decreases as Δf increases. When Δf is above 0.18 THz, η drops to 0. The experimentally measured curve of η versus Δf has a similar trend. The above results are different from previous works[9, 16], which demonstrates η increases monotonously with the increase of Δf. It may be due to the fact that η is affected by both t_{pp} and Δf in our work.

Based on the proposed theoretical model, we also investigated the relationship between the Δf and η by altering the coupling strength ($|J|_{max}$). The Δf of the converted waves mainly depends on the frequency difference of the resonance modes when the two resonators are in strong coupling and weak coupling state. Since the frequency splitting in the strong coupling state depends on $|J|_{max}$, we can tune the value of Δf by changing $|J|_{max}$.

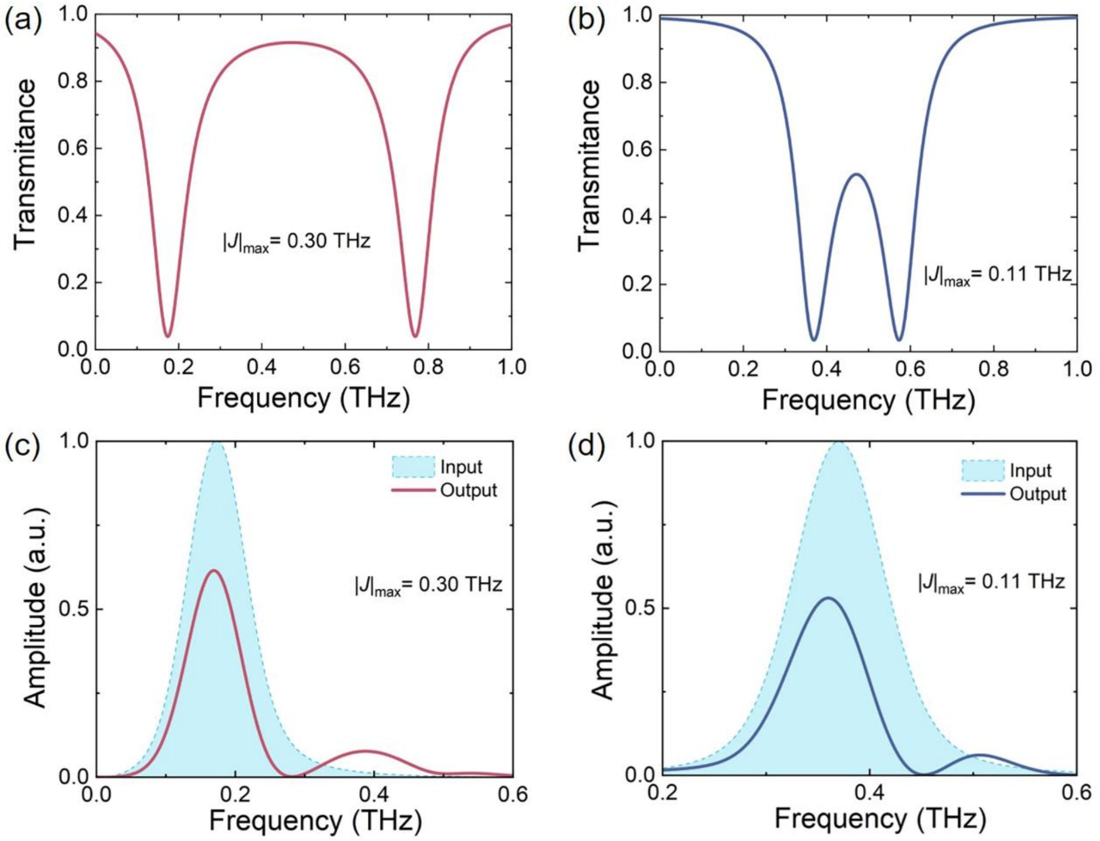

Fig. S17 Calculated transmission spectra when $|J|_{max}$ = 0.30 (a) and 0.11 THz (b), respectively. (c) Calculated power spectra of the input pulse with a center frequency of 0.17 THz and the output wave when $|J|_{max}$ = 0.30 and t_{pp} = -3.8 ps. (d) Calculated power spectra of the input pulse with a center frequency of 0.37 THz and the output wave when $|J|_{max}$ = 0.11 and t_{pp} = -3.8 ps.

In our calculation, we adjusted $|J|_{max}$ between 0.10 and 0.30 and calculated the frequency conversion effect. **Figure S17a, b** show the calculated transmission spectra when $|J|_{max}$ = 0.30 and 0.11, respectively. The frequency splitting between the two resonance dips of f_1 and f_2 when $|J|_{max}$ = 0.30 is remarkably larger than that when $|J|_{max}$ = 0.11. **Figure S17c, d** show the calculated power spectra of the input and output wave when $|J|_{max}$ = 0.30 and 0.11, respectively. It shows that the frequency conversion component is much higher when $|J|_{max}$ = 0.30.

The calculated η versus Δf under different $|J|_{max}$ when t_{pp} = -3.8 ps, is shown in Fig.S18. When $|J|_{max}$ is below 0.16, η decreases sharply with the decrease of $|J|_{max}$ while the decrease of Δf is very limited. When $|J|_{max}$ is higher than 0.16, η increases, and Δf

increases with increasing $|J|_{max}$. Based on our calculation results, we did not see a trend demonstrated in previous works [9, 16] that η increases monotonously with the increase of Δf. It may be because the resonance modes before and after the temporal boundary both have higher Q factors, and the value of η mainly depends on the contrast of the transmission spectra through the temporal boundary. The discrepancy also reflects that the working mechanism of our proposed device is different from previous work [9, 16].

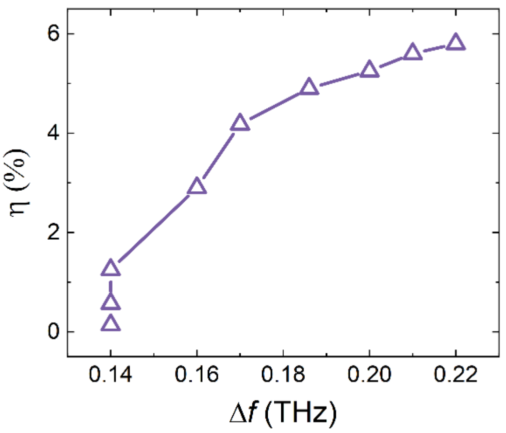

Fig. S18 Calculated η versus Δf under different $|J|_{max}$ when t_{pp} = -3.8 ps.

Supplementary References

1. Duan, S. Y., et al. Picosecond mode switching and Higgs amplitude mode in superconductor-metal hybrid terahertz metasurface. *Nanophotonics* **11**, 4253-4261 (2022).
2. Pérez-González, O., Zabala, N., Borisov, A. G., Halas, N. J., Nordlander, P., Aizpurua, J. Optical spectroscopy of conductive junctions in plasmonic cavities. *Nano Lett.* **10**, 3090-3095 (2010).
3. Zhang, C. H., et al. Active control of terahertz waves using Vanadium-Dioxide-Embedded metamaterials. *Phys. Rev. Appl.* **11**, 054016 (2019).
4. Zhang, H. F., et al. Experimental study on the transition of plasmonic resonance modes in double-ring dimers by conductive junctions in the terahertz regime. *Opt. Express* **24**, 27415-27422 (2016).
5. Wu, J. B., et al. Tuning of superconducting niobium nitride terahertz metamaterials. *Opt. Express* **19**, 12021-12026 (2011).
6. Hirori, H., Doi, A., Blanchard, F., Tanaka, K. Single-cycle terahertz pulses with amplitudes exceeding 1 MV/cm generated by optical rectification in $LiNbO_3$. *Appl. Phys. Lett.* **98**, 091106 (2011).

7. Ryusuke, M., Ryo, S. Nonequilibrium BCS state dynamics induced by intense terahertz pulses in a superconducting NbN film. *Phys. Rev. Lett.* **109**, 187002 (2012).

8. Hu, Y. W., et al. On-chip electro-optic frequency shifters and beam splitters. *Nature* **599**, 587-593 (2021).

9. Lee, K., et al. Linear frequency conversion via sudden merging of meta-atoms in time-variant metasurfaces. *Nat. Photonics* **12**, 765-773 (2018).

10. Zhang, C. H., et al. Terahertz nonlinear superconducting metamaterials. *Appl. Phys. Lett.* **102**, 081121 (2013).

11. Kang, L., et al. Suppression of superconductivity in epitaxial NbN ultrathin films. *J. Appl. Phys.* **109**, 033908 (2011).

12. Srivastava, Y. K., Manjappa, M., Krishnamoorthy, H. N. S., Singh, R. Accessing the high-Q dark plasmonic Fano resonances in superconductor metasurfaces. *Adv. Opt. Mater.* **4**, 1875-1881 (2016).

13. Khanikaev, A. B., Shvets, G. Two-dimensional topological photonics. *Nat. Photonics* **11**, 763-773 (2017).

14. Yin, S. X., Galiffi, E., Alù, A. Floquet metamaterials. *eLight* **2**, 8 (2022).

15. Chen, W. C., Mock, J. J., Smith, D. R., Akalin, T., Padilla, W. J. Controlling gigahertz and terahertz surface electromagnetic waves with metamaterial resonators. *Phys. Rev. X* **1**, 021016 (2011).

16. Lee, K., et al. Resonance-enhanced spectral funneling in Fabry–Perot resonators with a temporal boundary mirror. *Nanophotonics* **11**, 2045-2055 (2022).
